# Efficacy of a Plasmodium vivax Malaria Vaccine Using ChAd63 and Modified Vaccinia Ankara Expressing Thrombospondin-Related Anonymous Protein as Assessed with Transgenic Plasmodium berghei Parasites

**DOI:** 10.1128/IAI.01187-13

**Published:** 2014-03

**Authors:** Karolis Bauza, Tomas Malinauskas, Claudia Pfander, Burcu Anar, E. Yvonne Jones, Oliver Billker, Adrian V. S. Hill, Arturo Reyes-Sandoval

**Affiliations:** a The Jenner Institute, University of Oxford, Oxford, United Kingdom; b Division of Structural Biology, Wellcome Trust Centre for Human Genetics, University of Oxford, Oxford, United Kingdom; c Wellcome Trust Sanger Institute, Hinxton, Cambridge, United Kingdom

## Abstract

Plasmodium vivax is the world's most widely distributed malaria parasite and a potential cause of morbidity and mortality for approximately 2.85 billion people living mainly in Southeast Asia and Latin America. Despite this dramatic burden, very few vaccines have been assessed in humans. The clinically relevant vectors modified vaccinia virus Ankara (MVA) and the chimpanzee adenovirus ChAd63 are promising delivery systems for malaria vaccines due to their safety profiles and proven ability to induce protective immune responses against Plasmodium falciparum thrombospondin-related anonymous protein (TRAP) in clinical trials. Here, we describe the development of new recombinant ChAd63 and MVA vectors expressing P. vivax TRAP (PvTRAP) and show their ability to induce high antibody titers and T cell responses in mice. In addition, we report a novel way of assessing the efficacy of new candidate vaccines against P. vivax using a fully infectious transgenic Plasmodium berghei parasite expressing P. vivax TRAP to allow studies of vaccine efficacy and protective mechanisms in rodents. Using this model, we found that both CD8^+^ T cells and antibodies mediated protection against malaria using virus-vectored vaccines. Our data indicate that ChAd63 and MVA expressing PvTRAP are good preerythrocytic-stage vaccine candidates with potential for future clinical application.

## INTRODUCTION

Vaccines against the two most prevalent causative agents of human malaria, Plasmodium falciparum and Plasmodium vivax, remain elusive despite great efforts in their development. P. vivax is the most geographically widespread human malaria parasite, and it is considered to be the most prevalent form in some regions of Latin America and Central and Southeast Asia, accounting for 132 to 391 million clinical cases every year, and an estimated 2.85 billion people are considered at risk of P. vivax infection (1, 2).

Development of a vaccine against P. vivax malaria has been hindered by the lack of a method for long-term *in vitro* parasite culture in red blood cells (RBC) and by the requirement for suitable monkey challenge models, which are usually available only in their habitats. Despite significant progress in developing the necessary infrastructure (3–5), reliable studies in *Aotus* monkeys and humans are still limited to regions of endemicity, where the parasites can be collected from infected patients. Recently, the rodent malaria model Plasmodium berghei has been used to generate transgenic parasites expressing the ookinete surface protein P25 to assess a P. vivax transmission-blocking vaccine (6), an approach that has been extended to P. vivax antigens from preerythrocytic stages, such as the circumsporozoite protein (CSP) (7), and in this paper, we report the use of the thrombospondin-related anonymous protein (TRAP) expressed in transgenic P. berghei parasites.

TRAP mediates cell invasion in both the mosquito salivary glands and the hepatocytes of the vertebrate host (8), and currently, it is a leading malaria vaccine candidate that has shown efficacy through the induction of antigen-specific T cell responses in both animal models and humans (9–11).

In this study, heterologous prime-boost vaccinations using the clinically relevant recombinant chimpanzee adenovirus ChAd63 (Ad) and MVA viral vectors expressing the P. vivax TRAP (PvTRAP) transgene were assessed for immunogenicity and protection efficacy in various mouse strains, using a fully infectious transgenic P. berghei parasite carrying a perfect allelic replacement of the P. berghei TRAP (PbTRAP) gene with P. vivax TRAP. Our results indicate that protection was mediated not only by CD8^+^ T cells, but also by antibodies, which contrasts with previous findings for P. falciparum, where only T cells play a role in protection (11).

## MATERIALS AND METHODS

### Expression and purification of PvTRAP protein.

The codon-optimized gene of P. vivax TRAP (UniProt A5K806, residues Asp25 to Lys493) was cloned into the pHLsec vector (12) with a C-terminal hexahistidine tag (using primers 16 and 17 [see Fig. S1 in the supplemental material]). The protein was expressed by transient transfection in HEK-293T cells and purified from dialyzed (against phosphate-buffered saline [PBS] buffer) conditioned medium by immobilized Co^2+^ affinity chromatography, followed by size exclusion chromatography in 20 mM Tris-HCl, pH 8.0, 300 mM NaCl.

### Allelic-exchange vector for P. berghei TRAP.

The starting point for vector construction in Escherichia coli was a bacteriophage N15-based linear plasmid (13) from the *Plasmo*GEM resource (14; http://plasmogem.sanger.ac.uk) carrying a kanamycin resistance gene and an ∼8-kb genomic insert that included the P. berghei TRAP genomic locus (clone PbG01-2372c07). The genomic insert was modified in three steps by λ Red recombination (15) and a site-specific recombinase reaction (see Fig. S2 in the supplemental material), using protocols and reagents described previously (14, 16). We first designed a synthetic allele composed of the protein-coding sequence of P. vivax TRAP (accession number XM_001614097) from the Salvador I strain, which was codon optimized for expression in P. berghei using the GeneOptimizer software, which evaluates factors that can compromise mRNA stability, such as ribosomal binding sites, extreme GC content, cryptic splice and consensus sites, repeats, and secondary structures (Invitrogen, United Kingdom). The sequence was flanked with 50 bp of the 5′ untranslated region (UTR) and 600 bp of 3′ UTR sequences from PbTRAP. This 2.3-kbp construct was termed PvTR-trans and was obtained from GeneArt in a pMA plasmid backbone with a ColE1 origin and an ampicillin resistance marker. In step 1 (see Fig. S1a in the supplemental material), an attR1-zeo-pheS-attR2 cassette (16) was introduced 50 bp before the end of the 3′ UTR sequence in PvTR-trans. The first purpose of this cassette was to serve as a marker to exchange PbTRAP in clone PbG01-2372c07 for the synthetic PvTRAP allele under positive selection by zeocin (see Fig. S1b in the supplemental material). The *attR* sites then turned the bacterial marker into an exchange module for a P. berghei resistance marker in an *in vitro* Gateway reaction under negative selection by *p*-chlorophenylalanine against *pheS* (see Fig. S1c in the supplemental material) to generate the final targeting vector (see Fig. S1d in the supplemental material).

For Fig. S1a, E. coli bacteria carrying the pMA PvTR-trans plasmid were first made competent for recombination by transfecting plasmid pSC101gbaA-tet (17), carrying the bacteriophage λ *red* operon and E. coli *recA*. A PCR product generated with primers olSA00571F and olSA00572R (see Fig. S1 in the supplemental material) on plasmid pR6K attR1-zeo-pheS-attR2 was then transfected, and the recombination product was selected on zeocin as described previously (16) and cloned. E. coli bacteria harboring clone PbG01-2372c07 were rendered recombineering competent with pSC101gbaA-tet and electroporated with a PacI-SacI restriction fragment from the modified pMA plasmid composed of PvTR-trans, attR1-zeo-pheS-attR2, and the last 50 bp of the 3′ UTR of PbTRAP. Homologous recombination within the PbTRAP flanking sequences led to allelic exchange within the genomic DNA insert, and the product was selected on kanamycin and zeocin. The product of this reaction was then subjected to an *in vitro* Gateway reaction (Invitrogen) using the flanking *attR* sites to exchange zeo-pheS with a hDHFR-yFCU expression cassette for positive and negative selection in P. berghei, resulting in PbG01-2372c07-PvTRAP.

To obtain the donor plasmid for the Gateway reaction, we modified pR6K attL1-3xHA-hdhfr-yfcu-attL2 (14) as follows. First, a repeat of the *Pb*dhfr-ts 3′ UTR upstream of the hDHFR-yFCU cassette was eliminated as a PspOMI-FseI fragment, overhangs were removed using a Klenow fragment, and the vector was religated, resulting in pR6K-3xHA-noRR. Next, we inserted the first 500 bp of the PbTRAP 3′ UTR, which immediately follow the stop codon, into a unique Acc65I site within pR6K-3xHA-noRR downstream of the hDHFR-yFCU cassette, resulting in plasmid pR6K-3xHA-noRR500. The inserted 3′ UTR sequence, which was amplified from pMA-PvTR-trans using primers 14 and 15 (see Fig. S1 in the supplemental material), served as an upstream homology region to integrate the allelic-exchange vector into the P. berghei genome. It also duplicated the 3′ UTR already present in the PvTR-trans synthetic allele upstream of the hDHFR-yFCU cassette. Recombination between these directly repeated sequences eventually enabled the complete removal of all marker sequences from the transgenic parasite under negative selection against yFCU, leaving in the genome only the codon-adjusted synthetic allele, which had been generated without the need for restriction sites.

A NotI digestion released the P. berghei transfection vector from PbG01-2372c07-PvTRAP. The 5′ homology arm was 2.9 kb long, ensuring efficient homologous recombination and replacement of the TRAP allele. The 3′ homology arm was reduced to 0.5 kb as a result of a NotI site we included in primer 15 to eliminate the *attB2* site from the transfection vector, thereby preventing its integration into the P. berghei genome, where it would otherwise have remained after marker recycling.

### Parasite electroporation and clone selection.

Genetic modification of P. berghei was done by electroporation of ∼10^7^ purified schizonts from overnight cultures with 5 μg DNA. Electroporated merozoites were allowed to reinvade, at 37°C for 20 min *in vitro*, reticulocyte-rich blood from a donor mouse that had been pretreated 3 days earlier by intraperitoneal injection of 0.2 ml phenylhydrazine (6 mg/ml in PBS) to stimulate reticulocyte formation. The infected reticulocytes were then injected intraperitoneally into a naive mouse, and resistant parasites were selected during two passages by pyrimethamine supplied in the drinking water at 70 mg/liter. After the second passage, when the parasitemia reached 1%, parasites were dilution cloned by injecting 1 or 2 infected erythrocytes intravenously into each of 10 naive mice. To obtain allelic-exchange parasites that had lost the resistance cassette, infected mice were treated with flucytosine (5FC) prodrug at 0.5 mg/ml in drinking water for 4 or 5 consecutive days (18). Once the parasitemia reached 1%, dilutional cloning was repeated to obtain marker-free P. berghei clones expressing PvTRAP. For genotype analysis at different stages in the process, parasite genomic DNA was isolated from ∼1 ml of infected mouse blood obtained by cardiac puncture. White blood cells were removed on a column of CF11 cellulose powder (Whatman), and DNA was isolated using a QIAamp DNA Mini Kit (Qiagen).

### Animals.

Female inbred BALB/c (H-2^d^) and C57BL/6 (H-2^b^) and outbred CD1 (ICR) mice were used for the assessment of immunogenicity and protection after challenge. Tuck-ordinary (TO) outbred mice were used for parasite production and transmission. The mice were purchased from Harlan (United Kingdom).

### Ethics statement.

All animals and procedures were used in accordance with the terms of the United Kingdom Home Office Animals Act Project License. The procedures were approved by the University of Oxford Animal Care and Ethical Review Committee (PPL 30/2414).

### Parasite production.

Wild-type and transgenic parasites used to challenge mice were produced at the insectary of the Jenner Institute. Female Anopheles stephensi mosquitoes were fed on infected TO mice. Briefly, exflagellation was first confirmed, and mosquitoes were exposed to anesthetized infected mice for 10 min. The mosquitoes were then maintained for 21 days in a humidified incubator at a temperature of 19 to 21°C on a 12-h day-night cycle and fed with a fructose–*p*-aminobenzoic acid (PABA) solution.

### Viral vector vaccines.

Mice were primed with simian adenoviral vector 63 (ChAd63) encoding PvTRAP at a dose of 1 × 10^8^ infectious units (IU) and 8 weeks later boosted with modified vaccinia virus strain Ankara (MVA) carrying the same transgene at a concentration of 1 × 10^7^ PFU per ml, unless otherwise stated. All viral vector vaccines were administered intramuscularly in endotoxin-free PBS.

### Whole IgG ELISA.

Enzyme-linked immunosorbent assays (ELISAs) measuring total IgG were carried out as described previously (9), and serum antibody endpoint titers were taken as the *x* axis intercept of the dilution curve at an absorbance value 3 standard deviations greater than the optical density at 405 nm (OD_405_) for serum from a naive mouse.

### Peptides.

Crude 20-mer peptides overlapping by 10 amino acids and representing full-length P. vivax TRAP were synthesized by Mimotopes (Victoria, Australia). Individual peptide pools were used at a final concentration of 5 μg/ml.

### Ex vivo IFN-γ ELISPOT assay.

*Ex vivo* gamma interferon (IFN-γ) enzyme-linked immunosorbent spot (ELISPOT) assays were carried out using peripheral blood mononuclear cells (PBMCs) isolated from blood as previously described (10). MAIP ELISPOT plates (Millipore) were used to plate cells. Anti-mouse IFN-γ monoclonal antibody (MAb) and development reagents were used according to the manufacturer's specifications (Mabtech).

### Intracellular cytokine staining (ICS).

PBMCs were stimulated for 5 h in the presence of TRAP peptide pools as described above. Hepatic cellular responses were assessed from perfused livers that were digested with collagenase and treated with ammonium-chloride-potassium (ACK) buffer to lyse red blood cells. Phenotypic and functional analyses of CD8^+^ and CD4^+^ T cells were performed using the following antibody clones: anti-CD8 peridinin chlorophyll protein (PerCP)-Cy5.5 (clone 53-6.7), the nanocrystal eFluor 650-coupled anti-CD4 (GK1.5), anti-IFN-γ allophycocyanin (APC) (XMG1.2), anti-tumor necrosis factor alpha (TNF-α) eFluor 450 (MP6-XT22), and anti-interleukin 2 (IL-2) phycoerythrin (PE)-Cy 7 (JES6-5H4). Flow cytometric analyses were performed using an LSRII instrument (BD Biosciences, Oxford, United Kingdom). The frequencies of cells producing cytokines in the graphs represent data from which background from nonstimulated cells was subtracted. Data were analyzed with either FACSDiva or FlowJo software. Analysis of multifunctional CD8^+^ T cell responses was performed using Boolean analysis in FlowJo software, Pestle, and SPICE 4.0 (19).

### In vivo CD8^+^ and CD4^+^ T cell depletions.

T cell depletions were performed as described previously (20). Briefly, *in vivo* depleting MAbs were purified with protein G affinity chromatography columns from hybridoma culture supernatants. Anti-CD4 GK1.5 (rat IgG2a) and anti-CD8 2.43 (rat IgG2a) were sterile filtered and diluted in sterile PBS. Normal rat IgG (nRatIgG) was purchased from Sigma and purified by the same method. For depletion of CD4^+^ or CD8^+^ T cells, mice were injected intraperitoneally (i.p.) with 200 μg of the relevant MAb on days −2 and −1 before challenge and on the day of challenge.

### Whole IgG passive transfer.

Whole IgG from adenovirus-primed, MVA boost-immunized mice was purified using Pierce columns prepacked with 2 ml of protein G resin according to the manufacturer's instructions (Thermo Scientific, United Kingdom). Two milligrams of purified whole IgG obtained from immunized animals was injected into each naive CD1 mouse.

### Immunofluorescence assay (IFA).

Sporozoites isolated from mosquito salivary glands were loaded onto glass slides and allowed to air dry overnight at room temperature. The following day, the sporozoites were fixed in 4% paraformaldehyde and quenched with 0.01% sodium borohydride. The slides were then blocked for 1 h with 10% goat serum, 1% bovine serum albumin (BSA) in PBS before the addition of either 3D11 MAb specific to the repeat region of PbCSP (21) or anti-PbTRAP- and anti-PvTRAP-specific sera. Bound IgG was detected with goat anti-mouse IgG-Alexa 488 conjugate. Nuclear DNA was counterstained with 4,6-diamidino-2-phenylindole. The slides were viewed under a Leica DMI3000 microscope.

### Statistical model for parasitemia prediction.

To extrapolate the liver-to-blood parasite load or predict the time to 1% blood stage infection, a linear regression model was used as described previously (22). Briefly, blood parasite counts were obtained for 3 to 5 consecutive days starting on day 5 after challenge. Blood smears were stained with Giemsa stain, and percentages of parasitemia were calculated in all animals. The logarithm to base 10 of the calculated percentage of parasitemia was plotted against the time after challenge, and the Prism 5 for Mac OS X (GraphPad Software) statistical analysis package was used for generating a linear regression model on the linear part of the blood stage growth curve.

### Statistical analysis.

For all statistical analyses, GraphPad Prism version 5.0 for Mac OS X was used unless otherwise indicated. Prior to statistical analysis to compare two or more populations, the Kolmogorov-Smirnov test for normality was used to determine whether the values followed a Gaussian distribution. An unpaired *t* test was employed to compare two normally distributed groups, whereas a Mann-Whitney rank test was used for comparing two nonparametric groups. If more than two groups were present, nonparametric data were compared using a Kruskal-Wallis test with Dunn's multiple-comparison posttest, whereas normally distributed data were analyzed by one-way analysis of variance (ANOVA) with Bonferroni's multiple-comparison posttest. The effect of two variables was explored using two-way ANOVA with Bonferroni's multiple-comparison posttest. Correlation strength was tested using either Pearson's or Spearman's test, as indicated in Results. Kaplan-Meier survival curves were used to represent protective efficacy against a challenge with any P. berghei parasite lines. All ELISA titers were also log_10_ transformed before analysis. A *P* value of <0.05 was considered statistically significant.

## RESULTS

### An allelic-replacement P. berghei mutant expressing P. vivax TRAP.

Malaria vaccine candidates are often evaluated preclinically using Plasmodium species infecting laboratory rodents. For preclinical evaluation, experimental vaccines targeting a Plasmodium protein are often constructed against the orthologous protein of a rodent Plasmodium species to permit assessment of efficacy. However, efficacy levels in humans do not necessarily follow the same pattern as those in mice. We therefore chose to evaluate different experimental vaccines against P. vivax TRAP in mice infected with a transgenic P. berghei parasite, in which the endogenous PbTRAP gene was irreversibly replaced with its ortholog from P. vivax (PvTRAP). Using phage recombinase-mediated engineering of Plasmodium DNA in E. coli (14), we constructed an allelic-replacement vector that resulted in the introduction of the open reading frame (ORF) of PvTRAP flanked by entirely unaltered 3′ and 5′ UTR-flanking genomic sequences of P. berghei TRAP from the endogenous genomic locus (see Fig. S1a to d in the supplemental material). This strategy minimized the risk that expression of the transgene would be disturbed by engineered restriction sites or truncated UTRs, as has been observed with P. berghei CSP (23). The generation and selection of a transgenic P. berghei parasite line was made by positive selection with pyrimethamine, followed by the selection of parasites without the hDHFR-yFCU selection cassette by negative selection, resulting in the final transgenic line that contains the PvTRAP protein-coding region, which had previously been codon adjusted for expression in P. berghei (see Fig. S2a to e in the supplemental material). Since more transgenic rodent malaria lines are being generated by replacement or addition of a new gene, we refer to this transgenic parasite as PbANKA-PvTRAP(r)PbTRAP, in which the r stands for replacement.

### Assessment of the biological fitness of the transgenic parasite.

An allelic replacement of P. berghei TRAP with its P. falciparum ortholog (PfTRAP) has previously been reported to reduce the sporozoite load in salivary glands and sporozoite gliding; it also diminished the infectivity of intravenously injected sporozoites roughly 3-fold, as judged by a 12-h increase in the prepatency period (24). Therefore, we assessed the ability to infect mosquitoes and mice, as well as the proliferative capacity in both hosts, which we refer to as the “fitness” of PvTRAP transgenic parasites with respect to the wild type. Feeding mosquitoes on infected mice resulted in similar numbers of oocysts per mosquito (see Fig. S3a in the supplemental material). A small reduction in the average number of salivary gland sporozoites per mosquito was not statistically significant (*P* > 0.2 by Student's *t* test) (see Fig. S3a in the supplemental material), indicating similar abilities of the two parasites to infect salivary glands.

An IFA with polyclonal anti-PvTRAP antibodies confirmed the expression of PvTRAP on the surfaces of transgenic, but not wild-type, sporozoites, while PbTRAP was detected only on wild-type sporozoites (see Fig. S3b in the supplemental material). For comparison, we show that sporozoites of both parasite lines express the CS protein, as shown by staining with the 3D11 monoclonal antibody (21).

The ability of the PvTRAP transgenic parasites to infect the liver was investigated by intravenous administration of 1,000 salivary gland sporozoites into C57BL/6 mice. Mice injected with either transgenic or wild-type sporozoites developed parasitemia, and a linear regression analysis using the percentage of infected erythrocytes over three consecutive days indicated a slight (∼6-h) delay in reaching 1% parasitemia by the transgenic parasites compared to wild-type sporozoites (see Fig. S3c and d in the supplemental material). However, this difference was not statistically significant (*P* > 0.15 by Student's *t* test). Taken together, these data indicate that the allelic replacement of PbTRAP with PvTRAP did not diminish the parasite's ability to develop within A. stephensi mosquitoes or the vertebrate host.

### Immunogenicity of a heterologous prime-boost regimen using ChAd63-PvTRAP, followed by MVA-PvTRAP.

Two virus-vectored vaccines suitable for use in humans (25) were engineered to express PvTRAP. Both MVA-PvTRAP and the chimpanzee ChAd63-PvTRAP (Ad) were used to immunize inbred BALB/c and C57BL/6 and outbred CD1 mice. Cellular and humoral responses were quantified at various time points after applying a heterologous prime-boost regimen consisting of ChAd63-PvTRAP prime followed by a boost with MVA-PvTRAP, as reported previously (9).

**FIG 1:**
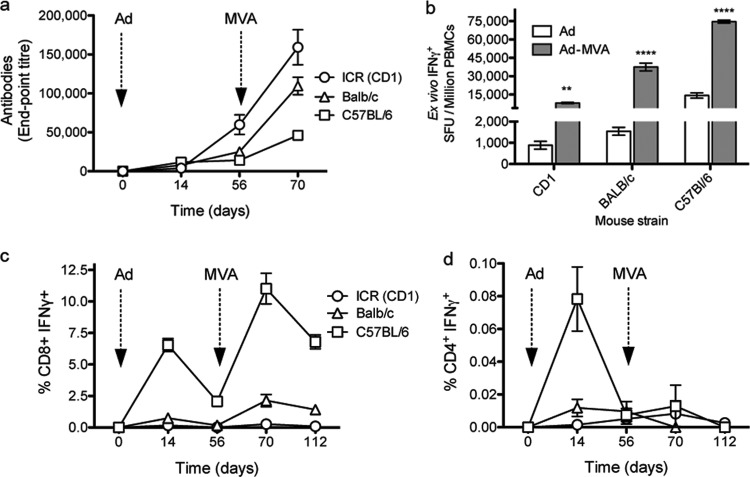
Kinetics of the humoral and cellular immune responses following immunization with PvTRAP. (a) Antibody titers for three mouse strains immunized using a heterologous AdCh63 MSA regime (Ad-MVA) were determined using an endpoint titer ELISA. (b) Antigen-specific T cell responses as measured by *ex vivo* IFN-γ ELISPOT assay 2 weeks after Ad prime or after Ad-MVA prime-boost. PBMCs were stimulated with three subpools of peptides overlapping the whole length of the PvTRAP protein. Nonspecific responses from unstimulated cells were subtracted to calculate the final number of SFU per million PBMCs. The frequencies of IFN-γ-producing CD8^+^ (c) and CD4^+^ (d) T cell responses were quantified by intracellular cytokine staining. The graphs show means and standard errors of the mean (SEM); *n* = 6 to 8 mice per strain. Statistical differences between prime and prime-boost regimens were calculated using ANOVA. Differences are indicated as follows: **, *P* < 0.01; ****, *P* < 0.0001.

**FIG 2:**
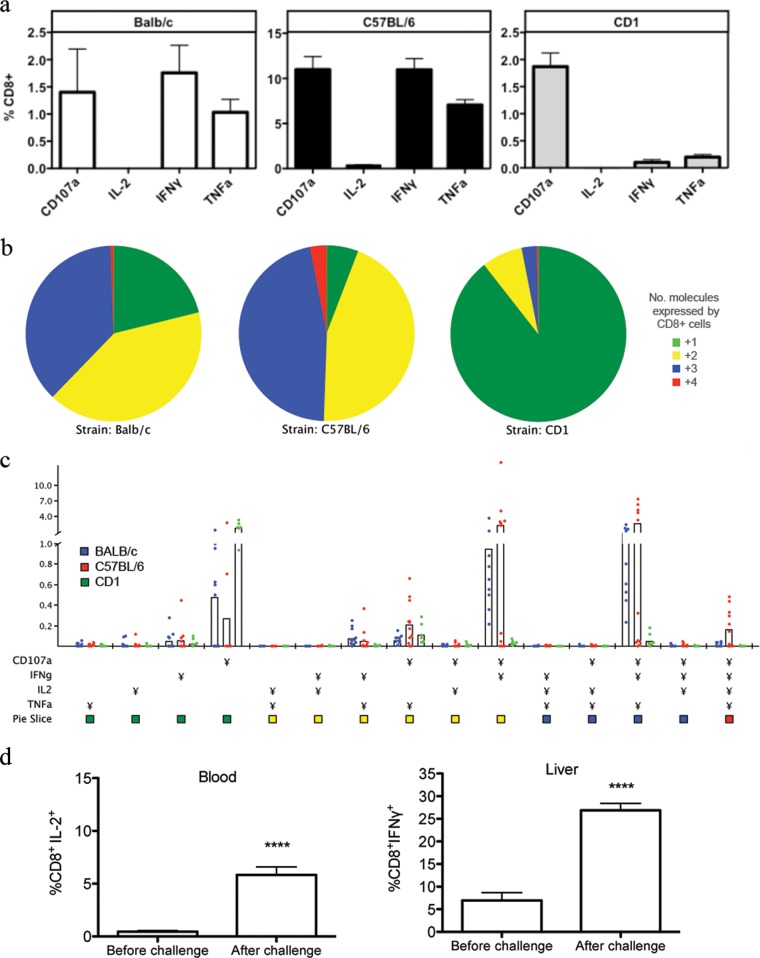
Multifunctional CD8^+^ T cell responses in mice immunized with Ad-MVA PvTRAP. The functionality of CD8^+^ T cells was measured in week 2 after the MVA boost. Cells were stimulated with a peptide pool representing the whole length of the PvTRAP protein. (a) Frequencies of CD8^+^ cells expressing CD107a, IL-2, TNF-α, and IFN-γ upon stimulation with PvTRAP. Background from nonstimulated wells was subtracted. The error bars indicate standard error of the mean (SEM). (b) Relative frequencies of cells expressing 4, 3, 2, or 1 marker protein. The functionality of PvTRAP-specific CD8^+^ cells was calculated using Pestle and SPICE software. (c) Multifunctionality of CD8^+^ cells measured as absolute frequencies of cells in peripheral blood mononuclear cells (PBMCs). (d) Production of IL-2 by CD8^+^ T cells upon infection with P. berghei sporozoites (left) and increase in frequencies of liver CD8^+^ T cells producing IFN-γ (right). Assessment of cytokine production was made on day 8 after an intravenous challenge with 1,000 sporozoites using BALB/c mice previously vaccinated with an Ad-MVA regimen. Control mice were not challenged. ****, *P* < 0.0001 calculated using a *t* test.

High antibody titers were induced after priming with the ChAd63-PvTRAP vector, and particularly after a ChAd63-PvTRAP prime, MVA-PvTRAP boost regimen in the outbred CD1 mouse strain, where titers of up to 159,000 were observed after the MVA-PvTRAP boost (Fig. 1a). High frequencies of T cell responses were also induced by the immunization, particularly in C57BL/6 mice, where a mean of 14,200 IFN-γ spot-forming units (SFU)/10^6^ PBMCs after ChAd63-PvTRAP prime and 75,000 IFN-γ SFU/10^6^ PBMCs after MVA-PvTRAP boost were elicited (Fig. 1b). Responses were assessed by ICS flow cytometry, and release of IFN-γ upon stimulation with a pool of peptides spanning the whole PvTRAP protein was predominantly within the CD8^+^ compartment, while production of IFN-γ by CD4^+^ T cells was minimal (Fig. 1c and d). ICS permits quantification of the production of various cytokines by a single cell, or multifunctionality, which has been used to correlate protection against infection with levels of functionality (9). Upon immunization, CD8^+^ T cells were able to express the proinflammatory cytokine TNF-α. The cells also produced IFN-γ, which plays an essential role in protection against malaria and is one of the major correlates of protection against the disease. CD8^+^ cells also upregulated CD107a (LAMP-1), which is a functional marker of degranulation and cytotoxicity, on their surfaces (Fig. 2a to c). IL-2 production by CD8^+^ cells was low after vaccination in all mouse strains. To determine if the cells were able to produce the cytokine and to obtain an indication of their ability to proliferate, vaccinated BALB/c mice were infected by intravenous administration of P. berghei sporozoites, and cytokine production was assessed 1 week after infection and compared to that of unchallenged controls. A significant increase in CD8^+^ cells producing IL-2 was detected in the blood of infected mice at the same time when frequencies of CD8^+^ T cells increased in the livers of infected mice, indicating the proliferative ability of CD8^+^ cells and the capacity to increase in numbers in the liver, where the parasite was proliferating (Fig. 2d).

### Breadth of the PvTRAP-specific T cell responses and immunodominant epitopes in mice.

**FIG 3:**
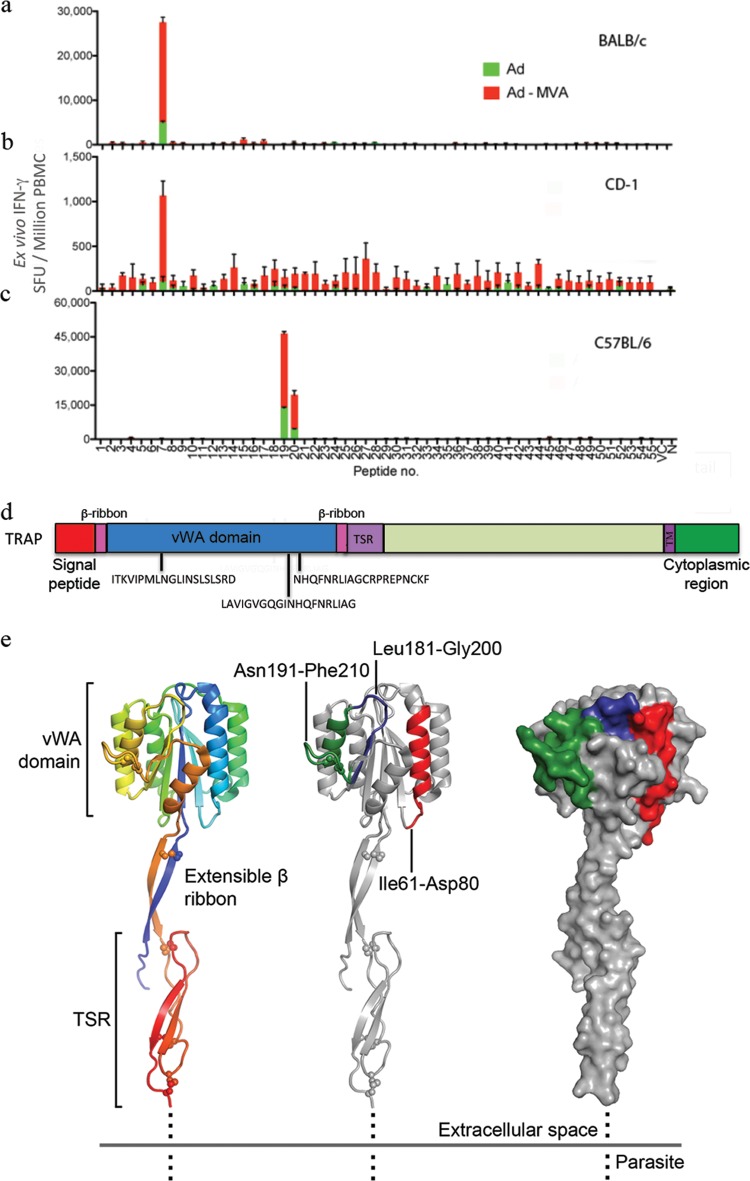
Breadth of the PvTRAP-specific T cell responses and immunodominant epitope mapping. (a to c) Groups of mice (*n* = 6) were immunized using an Ad-MVA PvTRAP regimen, and blood samples were taken 2 and 8 weeks after immunization to assess T cell responses using an *ex vivo* IFN-γ ELISPOT assay. Responses to individual peptides are shown for BALB/c, CD-1, and C57BL/6 mice. The error bars indicate SEM. (d) Schematic representation of PvTRAP indicating the functional domains of the molecule that were recognized by T cells. TSR, thrombospondin type 1 repeat domain; TM, transmembrane region; β-R, extensible β-ribbon. (e) Mapping of peptides onto the crystal structure of PvTRAP extracellular domain (26). (Left) Rainbow-colored cartoon diagram of PvTRAP (PDB ID 4HQO, residues Asp25 to Pro283), with N and C termini shown in blue and red, respectively. Domain regions are indicated and correspond to panel d. Disulfide bonds are shown as spheres. (Middle) Epitope-containing peptides 7 (Ile61 to Asp80), 19 (Leu181 to Ile190), and 20 (Asn191 to Phe210) map onto the vWA domain. (Right) The same peptides mapped onto the surface of the vWA domain form a continuous surface-exposed region.

Vaccination with recombinant Ad and MVA viral vectors expressing the PvTRAP antigen induced high frequencies of antigen-specific T cells, mainly from the CD8^+^ compartment. The immunodominant PvTRAP epitopes were further mapped in all inbred and outbred mouse strains tested with an *ex vivo* IFN-γ ELISPOT assay using individual 20-mer peptides for T cell stimulation. One immunodominant epitope was found in both BALB/c and CD1 mice, while in C57BL/6 mice, responses were induced by two adjacent peptides that share some amino acids, which raises the possibility of a single immunodominant epitope being present in both peptides (Fig. 3a to c). All immunodominant epitopes were restricted to the CD8^+^ compartment. Interestingly, when mapped onto the recently determined crystal structure of the PvTRAP ectodomain (26), peptides containing the immunodominant epitopes were all present on the Willebrand factor type A (vWA) domain (Fig. 3d).

### A ChAdPvTRAP-MVAPvTRAP vaccination regimen protects against challenge with P. berghei PvTRAP sporozoites.

**FIG 4:**
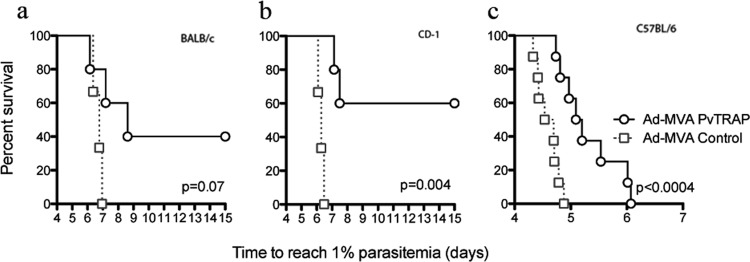
Protective efficacy of an Ad-M PvTRAP vaccination against a transgenic P. berghei parasite. BALB/c, CD1, and C57BL/6 mice were vaccinated with a heterologous prime-boost regimen using ChAd63-MVA viral vectors expressing PvTRAP. Two weeks after the final vaccination, the mice were challenged with 1,000 transgenic P. berghei PvTRAP sporozoites by intravenous injection. Parasitemia was monitored for 3 consecutive days starting from day 5 after challenge, and a model predicting the time to reach 1% parasitemia was generated. The absence of blood stage parasites in the animals that remained sterilely protected by day 8 was confirmed on day 15 postchallenge. A statistical analysis was performed using a Kaplan-Meier survival curve, and *P* values were calculated using Fisher's exact test.

The protective efficacy of a ChAd63-PvTRAP prime followed by an MVA-PvTRAP boost 8 weeks apart was assessed by challenge of the immunized mice with 1,000 transgenic sporozoites expressing PvTRAP. Protective efficacy varied between mouse strains, and complete, sterile protection was 40% in BALB/c and 60% in CD1 mice (Fig. 4 a and b). In view of the low (antigen-specific) CD8^+^ T cell responses, the high level of protective efficacy was surprising; we compared it to C57BL/6 mice, where the magnitude of CD8^+^ T cell responses was higher but there were no sterilely protected animals (Fig. 4c). We have observed for a number of years that the C57BL/6 mouse strain is highly sensitive to malaria induced by a P. berghei sporozoite challenge, and our recent results support this observation. Nevertheless, having mouse strains with different susceptibilities to malaria could provide a good tool to explore vaccines with various levels of protection, and C57BL/6 mice could permit the identification of highly protective vaccines.

### Antibodies and CD8^+^ cells as protective mechanisms against a transgenic sporozoite challenge.

**FIG 5:**
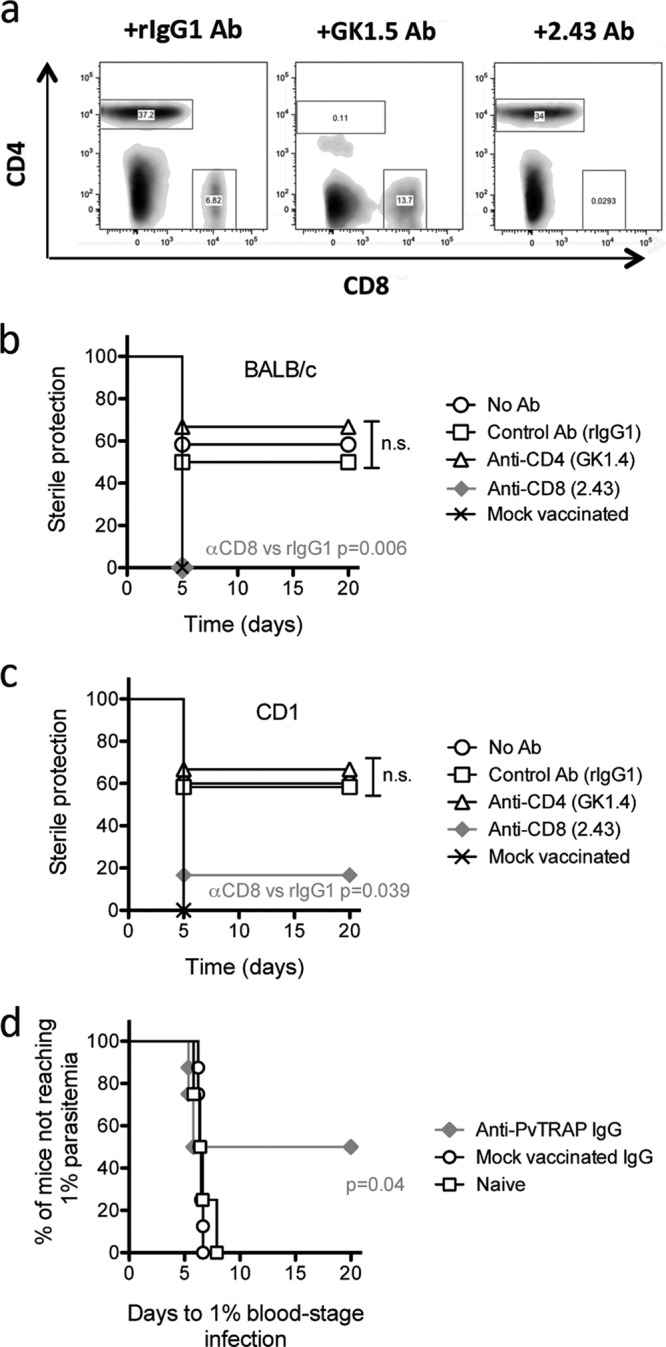
Protection against sporozoite challenge is mediated by both PvTRAP-specific CD8^+^ T cells and antibodies. (a) Flow cytometry analysis of PBMCs from mice, indicating that injection of anti-GK1.5 or anti-2.43 was approximately 99% effective at depleting CD4^+^ or CD8^+^ cells, respectively. rlgG1 MAb served as a negative control. (b and c) Effect of depletion of CD4^+^ or CD8^+^ T cells on protection against parasite challenge. BALB/c and CD1 mice (*n* = 12) were first vaccinated using a heterologous prime-boost regimen with Ad-MVA expressing PvTRAP. Two weeks after the final vaccination, the mice received depleting anti-GK1.5 or anti-2.43 antibodies on days −2, −1, and 0 relative to the challenge. *P* values were generated using a Kaplan-Meyer method to compare survival curves. n.s., not significant. (d) Survival curve of CD1 mice upon PvTRAP antibody transfer. Two weeks after the last vaccination, whole IgG was purified from serum, and 2 mg was transferred to naive CD1 mice (PvTRAP IgG). Whole IgG raised against empty viral vectors was also included (control IgG).

We further investigated the immune mechanisms associated with protection in a PvTRAP transgenic sporozoite challenge. CD4^+^ or CD8^+^ T cells were depleted by administration of the monoclonal antibody GK1.5, which binds to the CD4^+^ molecule, or the monoclonal antibody 2.43, which binds to the CD8^+^ molecule, both of which induce the depletion in blood of cells bearing these coreceptors (Fig. 5a). Antibodies were administered on three consecutive days after a boosting vaccine with MVA-PvTRAP, which corresponded to days −2, −1, and 0 with respect to the time of the sporozoite challenge.

Depletion of CD4^+^ T cells did not affect protection against sporozoite infection in BALB/c or CD1 mice (Fig. 5b and c). However, depletion of CD8^+^ cells with the 2.43 MAb significantly reduced protection in both mouse strains (Fig. 5b and c). Importantly, despite the complete CD8^+^ depletion, 17% of the CD1 mice were still sterilely protected, an indication that perhaps other factors could play a role in protection against malaria sporozoite stages (Fig. 5c).

Therefore, we investigated the role of PvTRAP-specific antibodies in protection against malaria by transferring purified IgG from immunized CD1 mice into naive recipient mice that were subsequently challenged with the transgenic PvTRAP parasite. Fifty percent of CD1 mice that had received the anti-PvTRAP antibodies were sterilely protected (*P* = 0.04 by Fisher's exact test), while all naive mice and the controls injected with IgG from empty-vector-immunized mice succumbed to infection (Fig. 5d). This result indicated that anti-PvTRAP antibodies mediate protection, in addition to the antigen-specific CD8^+^ T cells.

## DISCUSSION

P. vivax is responsible for the most widespread form of malaria in the world. We report a newly developed preerythrocytic vaccination regime using two clinically relevant virus-vectored vaccines expressing PvTRAP (MVA-PvTRAP and ChAd63-PvTRAP). Development of malaria vaccines, including P. vivax malaria, has been hindered by numerous factors. Among them, none of the human malaria sporozoites infect mice, and therefore, the easily accessible rodent models cannot be used to test the protective efficacy of a new vaccine. For this reason, the assessment of vaccine efficacy has relied on the use of mouse and primate models, in which animals are infected by other malaria parasites that can differ in the sequence or presence of genes and proteins that are tested as vaccine candidates.

To enable the assessment of vaccine efficacy in a preclinical model, we constructed a transgenic P. berghei parasite expressing PvTRAP as a perfect allelic replacement for the PbTRAP gene that showed normal infectivity in mice. Our strategy minimized the risk of exogenous sequence or restriction sites affecting the sporozoite fitness of the transgenic parasites, as has been reported for transgenic P. berghei parasites in which the PbCSP gene was replaced by the CSP gene of P. falciparum and that contained a selection marker cassette in the mutated CSP locus (27). We propose that a similar allelic-replacement strategy could become a standard approach in the assessment of vaccine candidates when evaluated by preclinical testing in rodent malaria models. It could further help to define the correlates needed for protection, such as the quality or quantity of antibodies and T cells required to protect against malaria, and permit easier validation of new vaccines while reducing the need for parasite challenge studies in nonhuman primates.

Mapping of the immunodominant epitopes on the PvTRAP crystal structure revealed that CD8^+^ responses were directed toward the vWA, and depletion of such CD8^+^ cells decreased protection substantially. This is in agreement with early studies in mice that established the role of CD8^+^ T cells in protection against SSP2 (TRAP) (28, 29) and previous clinical trials using P. falciparum TRAP, where protection was associated with CD8^+^ T cells in the absence of antibodies (11). Nevertheless, our results indicated that transfer of antibodies from PvTRAP-immunized mice into naive recipients also contributed to protection. Reports have shown that in regions where malaria is endemic there are TRAP-specific antibodies in the blood of patients that correlate with protection (30) and that immunization of humans and mice with irradiated sporozoites can induce protective antibody responses against SSP2 (TRAP) and inhibit sporozoite invasion of human hepatoma cells (28, 31, 32). Crystal structures determined recently show PfTRAP and PvTRAP in two distinct conformational states, closed and open, respectively (26). This raises the possibility that structural differences between the TRAP molecules of two different species may contribute to distinctive TRAP-antibody interactions, as antibody recognition could rely on conformational epitopes.

In summary, this study describes a highly immunogenic P. vivax candidate vaccine that can induce protection in mice against a transgenic parasite expressing the PvTRAP transgene. ChAd63 and MVA both yielded good immunogenicity and protective efficacy with P. falciparum TRAP in mouse models (9), which were highly predictive of good immunogenicity in rhesus macaques and humans (25, 33). The ChAd63-MVA prime-boost regimen for vaccination with PvTRAP, therefore, warrants further assessment in Aotus lemurinus griseimembra monkeys (3, 34) and humans.

## Supplementary Material

Supplemental material
