# Supplementary material for: Efficacy of a Plasmodium vivax Malaria Vaccine Using ChAd63 and Modified Vaccinia Ankara Expressing Thrombospondin-Related Anonymous Protein as Assessed with Transgenic Plasmodium berghei Parasites
Source: Infect Immun. 2014 Mar;82(3):1277–86. doi: 10.1128/IAI.01187-13 (PMC3957994; doi:10.1128/IAI.01187-13)
Supplement: Supplemental material [file supp_82_3_1277__index.html]

Efficacy of a Plasmodium vivax Malaria Vaccine Using ChAd63 and Modified Vaccinia Ankara Expressing Thrombospondin-Related Anonymous Protein as Assessed with Transgenic Plasmodium berghei Parasites — Supplemental material 

# Efficacy of a Plasmodium vivax Malaria Vaccine Using ChAd63 and Modified Vaccinia Ankara Expressing Thrombospondin-Related Anonymous Protein as Assessed with Transgenic Plasmodium berghei Parasites

## Supplemental material

**Files in this Data Supplement:**

- Supplemental file 1 -

  Fig. S1. Generation of an allelic exchange vector for PbTRAP by recombinase-mediated engineering in *E. coli*. Fig. S2. Generation of marker-free transgenic *P. berghei* parasites expressing *P. vivax* TRAP. Fig. S3. Fitness and phenotype analyses of a *P. berghei* PvTRAP clone.

  PDF, 5.1M
