# Supplementary material for: Efficacy of a Plasmodium vivax Malaria Vaccine Using ChAd63 and Modified Vaccinia Ankara Expressing Thrombospondin-Related Anonymous Protein as Assessed with Transgenic Plasmodium berghei Parasites
Source: Infect Immun. 2014 Mar;82(3):1277–86. doi: 10.1128/IAI.01187-13 (PMC3957994; doi:10.1128/IAI.01187-13)
Supplement: Supplemental material [file IAI.01187-13_zii999090566so1.pdf]

Supplementary Text and Figures

Figure S1: Generation of an allelic exchange vector for PbTRAP by recombinase mediated engineering in *E. coli*. (a) An attR1-zeo-pheS-attR2 cassette was incorporated downstream of a synthetic PvTRAP-trans gene by recombineering during the initial stage of cloning. **(b)** The resulting plasmid pMA-PvTR-trans-ZEO was digested at PacI and SacI sites and the resulting fragment incorporated by double homologous recombination into the genomic library clone replacing the PbTRAP gene. **(c)** Next, the *zeo-pheS* was exchanged for *hDHFR-yFCU* cassette in a site-specific recombinase reaction in the third step of cloning. **(d)** The final construct was digested at NotI sites releasing the integration cassette containing the PvTRAP gene, which on both sides was flanked by homology arms corresponding to the 5' and 3' UTR of PbTRAP.

Figure S2: Generation of marker-free transgenic *P. berghei* parasites expressing *P. vivax* TRAP. (a) Linear plasmid containing the allelic exchange vector flanked by NotI sites. **(b)** Schematic of the NotI fragment replacing PbTRAP by ends-out homologous recombination with the TRAP genomic locus. **(c)** Modified TRAP genomic locus after pyrimethamine selection. **(d)** Schematic illustration of marker removal by negative selection with 5-fluorocytosine prodrug. **(e)** Agarose gel showing PCR genotyping of clones obtained after initial pyrimethamine selection (KB1-m1) and after removal of the marker (KB1-m1-cl4). Primer annealing sites and sizes of expected products are shown in panels b-c. Oligonucleotide sequences are as shown in Table S1.

Figure S3: Fitness and phenotype analyses of a *P. berghei* PvTRAP clone. (a) Mean numbers of oocysts and salivary gland sporozoites in *Anopheles stephensi*

mosquitos 10 and 21 days, respectively, after an infectious feed. Error bars show standard deviation. Data was obtained from 4 separate experiments each analysing 40-50 mosquitoes per parasite line. **(b)** Immunofluorescence micrographs of sporozoites stained with species specific anti-TRAP antibodies or with mAb 3D11 against CSP. **(c)** Kaplan-Meier plot for “Time to 1% parasitaemia” (Tto1) in 8 C57BL/6 mice injected either with 1000 transgenic or wild type salivary gland sporozoites. **(d)** Average Tto1 predicted from a linear regression model. Mean with SEM are plotted and a Student’s t-test was used where relevant.

Figure S1

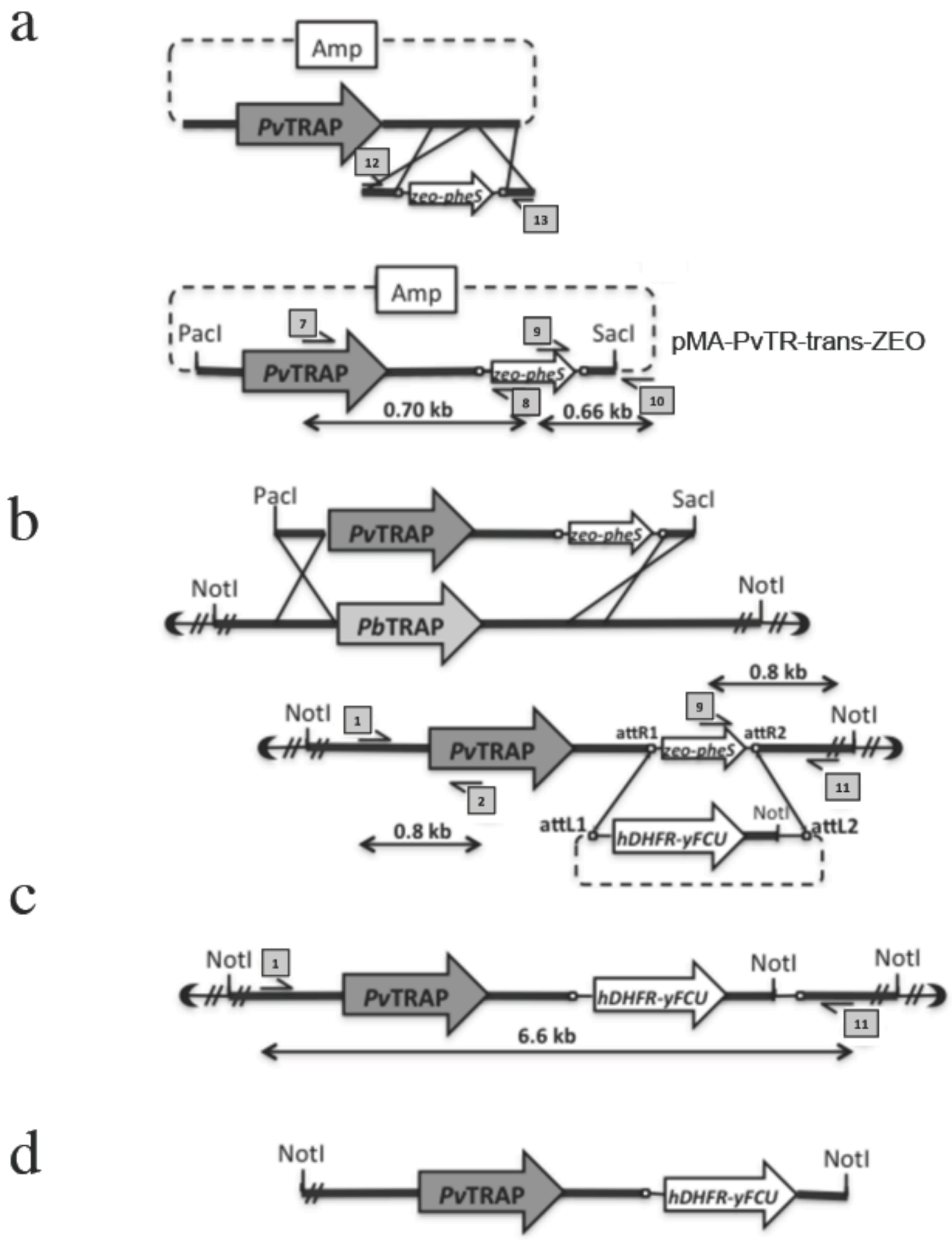
a
Amp
PvTRAP
12
zeo-pheS
13
Amp
PacI
7
9
SacI
pMA-PvTR-trans-ZEO
PvTRAP
zeo-pheS
8
10
0.70 kb
0.66 kb
b
PacI
PvTRAP
zeo-pheS
SacI
NotI
PbTRAP
NotI
0.8 kb
NotI
1
PvTRAP
9
attR1
zeo-pheS
attR2
NotI
2
11
NotI
attL1
hDHFR-yFCU
attL2
0.8 kb
c
NotI
1
PvTRAP
hDHFR-yFCU
NotI
NotI
11
6.6 kb
d
NotI
PvTRAP
hDHFR-yFCU
NotI

Figure S2

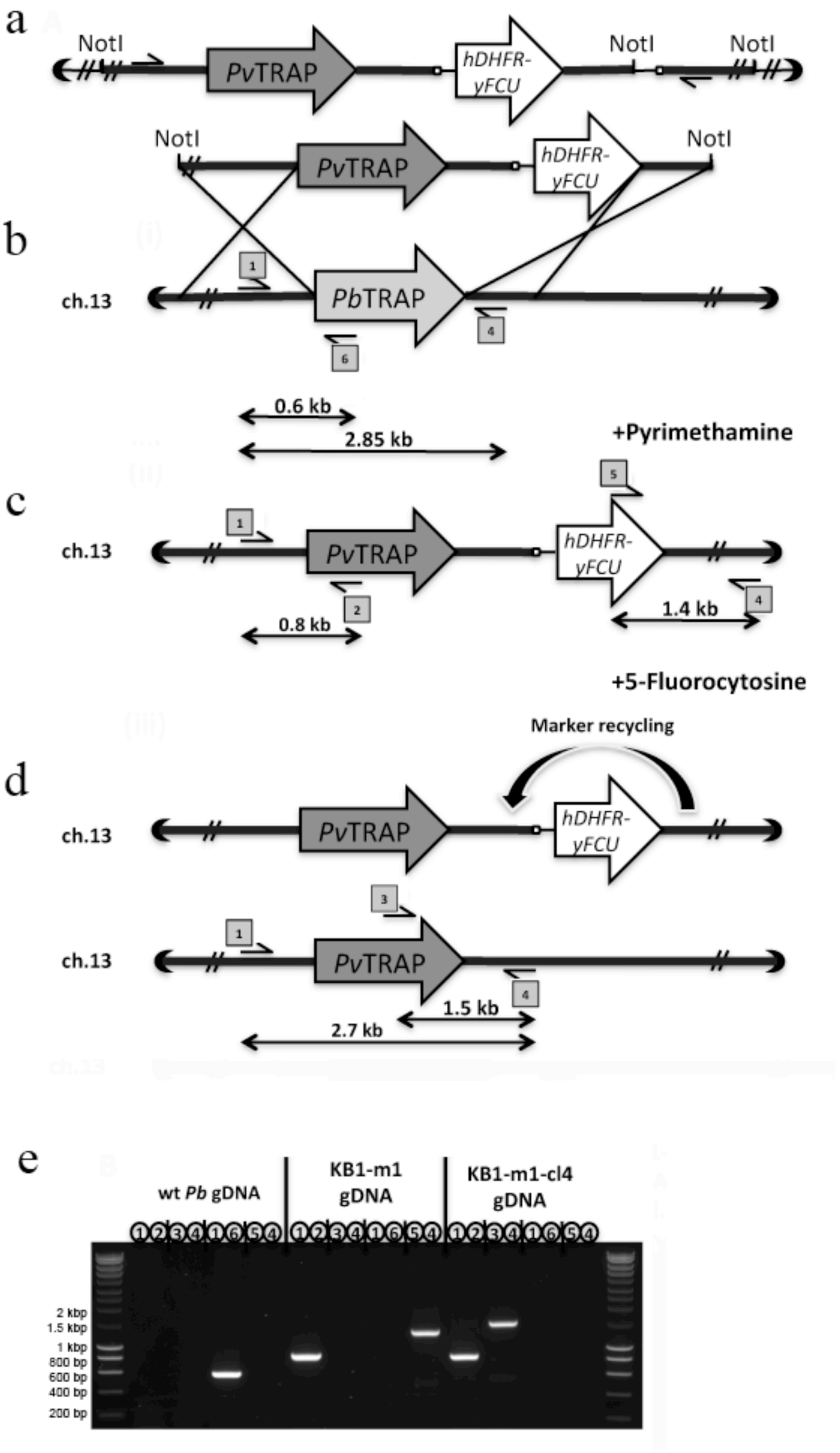

a
NotI
PvTRAP
hDHFR-yFCU
NotI
NotI
b
NotI
PvTRAP
hDHFR-yFCU
NotI
ch.13
PbTRAP
1
4
6
0.6 kb
2.85 kb
+Pyrimethamine
c
ch.13
1
PvTRAP
2
0.8 kb
5
hDHFR-yFCU
4
1.4 kb
+5-Fluorocytosine
Marker recycling
d
ch.13
PvTRAP
hDHFR-yFCU
ch.13
1
3
PvTRAP
4
1.5 kb
2.7 kb
e
wt Pb gDNA
KB1-m1 gDNA
KB1-m1-cl4 gDNA
2 kbp
1.5 kbp
1 kbp
800 bp
600 bp
400 bp
200 bp

Figure S3

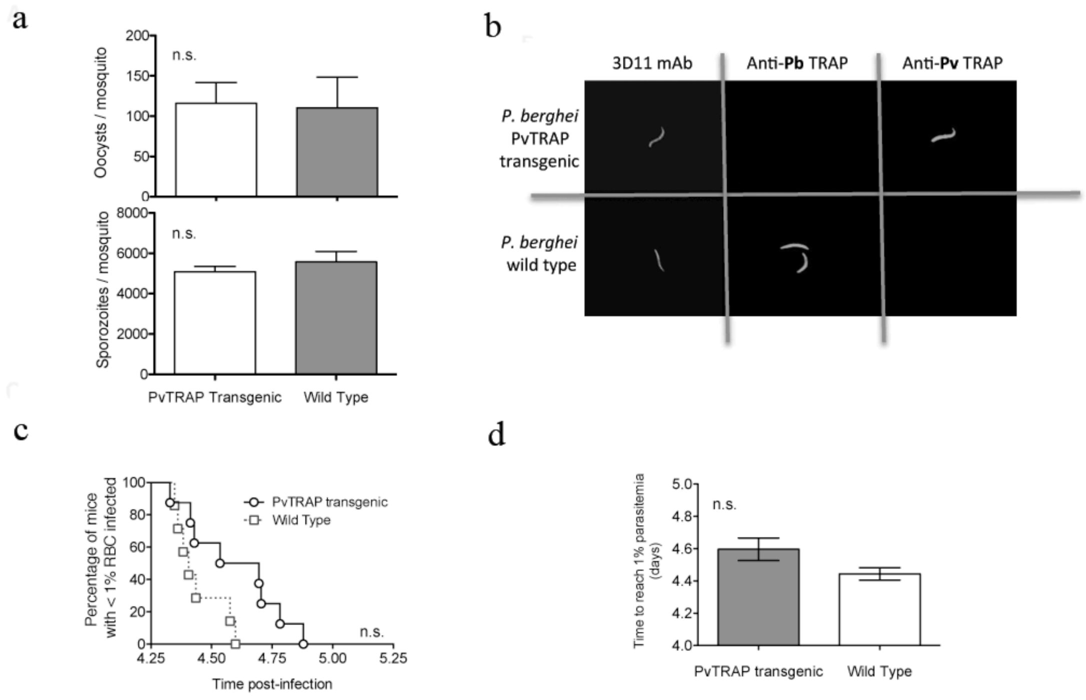
a
Oocysts / mosquito
n.s.
200
150
100
50
0
Sporozoites / mosquito
n.s.
8000
6000
4000
2000
0
PvTRAP Transgenic
Wild Type
b
3D11 mAb
Anti-Pb TRAP
Anti-Pv TRAP
P. berghei PvTRAP transgenic
P. berghei wild type
c
Percentage of mice with < 1% RBC infected
100
80
60
40
20
0
PvTRAP transgenic
Wild Type
n.s.
4.25
4.50
4.75
5.00
5.25
Time post-infection
d
Time to reach 1% parasitemia (days)
n.s.
5.0
4.8
4.6
4.4
4.2
4.0
PvTRAP transgenic
Wild Type
